# Cyr61 Mediates Angiotensin II-Induced Podocyte Apoptosis via the Upregulation of TXNIP

**DOI:** 10.1155/2023/8643548

**Published:** 2023-03-29

**Authors:** Jingxue Ma, Ruiying Ma, Xiaoyue Zhao, Yingxiu Wang, Shengxue Liao, Cong Nong, Fengling Lu, Zhao Liang, Junhui Huang, Yan Huang, Zhengxi Zhu, Junjie Wang

**Affiliations:** ^1^Department of Nephrology, The People's Hospital of Baise, Baise, China; ^2^Department of Nephrology, The Affiliated Hospital of Youjiang Medical University for Nationalities, Baise, China; ^3^Institute of Science and Technology Information in Baise, China; ^4^Department of Hematology, The People's Hospital of Baise, Baise, China; ^5^Department of Nephrology, Cangxi People's Hospital, Cangxi, Sichuan, China

## Abstract

**Purpose:**

It is well documented that angiotensin II (Ang II) elevation promotes apoptosis of podocytes *in vivo* and *vitro*, but the potential mechanism is still oscular. The current study is aimed at probing into the assignment of cysteine-rich protein 61 (Cyr61) in Ang II-induced podocyte apoptosis.

**Methods:**

Podocytes were treated with Ang II (10^−6^ mol/L) for 48 hours to establish an injury model in vitro. Western blot assays were detected the expression of Cyr61, Cyt-c, Bax, and Bcl-2. Gene microarray was used to analyze the expression of mRNAs after treatment with Ang II. CRISPR/Cas9 technology was used to knock down Cyr61 and overexpress TXNIP gene, respectively.

**Results:**

The expression of Cyr61, TXNIP, Cyt-c, and Bax in podocytes treated with Ang II were upregulated, but the expression and apoptotic rates of Bcl-2 in podocytes were inhibited. The level of the above factors was not significantly different after the knockdown of Cyr61 with Ang II in podocytes. In Ang II group, when knocked down Cyr61, the expressed level of TXNIP, Cyt-c, and Bax was diminished after Ang II treatment; interestingly Bcl-2 expression and podocyte apoptotic rate were reduced. Under the stimulation of Ang II, the expression of Cyt-c and Bax were growing, whereas Bcl-2 was reduced, and the apoptotic rates were higher in the TXNIP overexpression group. Cyt-c and Bax were put on, whereas that of Bcl-2 was to be cut down when the Cyr61 was knockdown, and the apoptotic rates were gained in the TXNIP overexpression+Cyr61 knockdown group.

**Conclusions:**

The results of the study extrapolate that Cyr61 plays a dominant role in Ang II-induced podocyte apoptosis. Additionally, Cyr61 may mediate the Ang II-induced podocyte apoptosis by promoting the expression of TNXIP.

## 1. Introduction

Podocytes enclose glomerular capillaries in the Bowman's capsule of the kidney, also called epithelial cells of the visceral layer of the kidney. They have been termed after the “peduncle” that protrudes from cells and are the most structurally complex and easily damaged terminal differentiated cells [1, 2]. Podocytes are an indispensable component of the glomerular filtration barrier which play an instrumental part in maintaining glomerular filtration function [3]. The occurrence and progression of multiple glomerular diseases involve damage to podocytes, and numerous recent studies have established that damage and loss of podocytes is one of the common clinical manifestations of glomerular diseases, including diabetic nephropathy (DN), microdegenerative nephropathy, focal stage glomerulosclerosis, membranous nephropathy, and lupus nephritis, and is one of the main causes of glomerular proteinuria [4]. Given that renal diseases are asymptomatic in the early stages, frequently develop chronically, and have persistent irreversible lesions, early prevention, control, and timely intervention are crucial to prevent progression into the uremic phase. The traditional concept is that some podocyte diseases are closely related to autoimmune dysfunction, and some patients develop hormone resistance and relapse after receiving conventional treatments such as immunosuppressive drugs and glucocorticoids. Therefore, an in-depth investigation is warranted to elucidate the mechanism underlying podocyte injury and the development of podocytosis, as well as provide a rationale for their clinical diagnosis and medical attendance.

Renin-angiotensin system (RAS) is one out of the earliest and most widely researched atypical hormone system reference several pathophysiological processes [5, 6]. It not only regulates blood pressure and internal environment homeostasis by acting on the renal and cardiovascular systems but is also implicated in other pathological processes, including inflammation and immune response [7]. RAS is crucial for the regulation of renal homeostasis, and inhibiting RAS in chronic kidney disease patients can minimize proteinuria and restore renal function. Recent studies have uncovered that the main effector molecules of RAS, including Ang II, salt corticosteroid, and protein receptors, are secreted and generated by podocytes secreted and main effect molecules of RAS, including Ang II, salt corticosteroids, and protein receptors. Furthermore, RAS is activated in numerous glomerular diseases, while their suppression plays a protective role against injury. Ang II, as one of the significant intermediates of RAS, plays a crucial role in podocyte injury. Several research projects have approved the probability of Ang II promoting podocyte apoptosis through various pathways [8–12]. In addition, Ang II can also trigger autophagy in podocytes [13]. However, it is needed to further explore the mechanism of Ang II-induced podocyte apoptosis. Exploring the local RAS of podocytes and the transduction mechanism of their activation may yield novel therapeutic targets for preventing podocyte injury.

Herein, gene microarray technology was used to analyze changes in mRNA expression after Ang II intervention in podocytes. Besides, the role of Cyr61 and TXNIP in Ang II-induced podocyte apoptosis was assessed by knocking down Cyr61 and overexpressing TXNIP with CRISPR/Cas9 technology.

## 2. Materials, Reagents, and Methods

### 2.1. Experimental Materials

#### 2.1.1. Materials and Reactants

Cell lines were purchased from the cell bank of Fudan University, Shanghai, and frozen in the Scientific Experiment Center of the Right River School of Ethnic Medicine; rabbit anti-Cyr61 antibodies (Santa Cruz Biotechnology); rabbit anti-Akt antibody, rabbit anti-Bax antibody, and rabbit anti-Bcl-2 antibody (Abcam); rabbit anti-TNXIP antibody (Cell Signaling); mouse anti-Cyt-c (Biyuntian); GAPDH (Beijing Zhongsun Jinqiao Biological Company); horseradish peroxidase-labeled goat anti-rabbit and goat anti-mouse secondary antibodies (Biyuntian); apoptosis detection kit (BD); Ang II (Solabao); protease inhibitor mixture (Kangwei Century); and BCA protein quantification kit (Biyuntian); murine Cyr61KO plasmid, murine TXNIP activation plasmid (VDUP1 activation plasmid), HDR transfection plasmid, and Ultracruz transfection reagent (Santa Cruz Biotechnology).

### 2.2. Experimental Methods

#### 2.2.1. The Cells Were Cultured and Differentiated according to a Previous Study [14]

The cells were cultured in RPMI-1640 nutrient medium supplemented with 10% fetal bovine serum at 37°C and 5% CO_2_. We replaced the medium every 1 to 2 days. When cell confluency reached 80%, they would be subsequently digested, passaged, and then used for the ensuing experiments after about 14 days of differentiation and maturation.

Gene microarray analysis: in order to detect alterations in mRNA expression following Ang II intervention in the pedunculated cells, the cells were collected, and total RNA was extracted after treating with Ang II (10-6 mol/L) after 48 h intervention in the pedunculated cells, and then, total RNA gene expression profile was analyzed using gene microarray technology.

#### 2.2.2. Cell Transfection

The cells were plated in six-well plates at a specific mass of 2 × 10^5^ cells/mL per orifice, and serum-free culture medium was replaced when the cell confluency reached 70%. Next, 1.0 *μ*g Cyr61 CRISPR/Cas9 KO plasmid and 1.0 *μ*g HDR plasmid were added to a 150 *μ*L serum-free antibiotic-free medium, thoroughly stirred and fostered for 5 min at indoor temperature subsequently. 10 *μ*g L Ultracruz transfection reagent was infunded to 140 *μ*L of serum-free and antibiotic-free substrate and thoroughly blended and incubated for 5 min at ambient temperature. The above two hybrids were blended and cultivated for 10 min at interior temperature, after which an antibiotic-free medium containing 10% FBS was fed into making the final volume 2 mL in a six-well plate containing the cells. Transfection was visualized under a fluorescence microscope at 24 h and 48 h. After 48-96 h, the cells were transfected with 8 *μ*g/mL puromycin, and the transfected cell lines were successfully established. The process for TXNIP transfection was identical to that for Cyr61.

#### 2.2.3. Detection of Apoptosis

After the podocytes were cultured for 48 h, the supernatant was discarded, and then, the cells in each group were washed with PBS at 4°C for 2 times. After trypsin digestion, the supernatant was combined with the above-mentioned supernatant to make single cell suspension, and the cell density was adjusted to 1 × 10^6^ cells/mL. 100 *μ*L cell suspension was decanted to 5 *μ*L Annexinv-FITC and 5 *μ*L PI dissolution, and the cells were reared at room temperature without light for 15 min. Then, 400 *μ*L PBS was added, and flow cytometry was used for detection of apoptosis.

#### 2.2.4. Detection of the Protein Expression Levels of Cyr61, TXNIP, Cyt-c, Bax, and Bcl-2 in Each Group via Western Blotting

The total protein of the glomerular podocytes line was extracted after being cultured for 48 h according to the previous grouping, and the protein concentration was detected using a BCA Kit. Following SDS-PAGE electrophoresis, the membranes were transferred at a constant voltage of 100 V for 110 min. After the transfer of the proteins to the NC membrane, the membrane was confined with a blocking buffer for 30 min and incubated with Cyr61, TXNIP, Cyt-c, Bax, and Bcl-2 primary antibodies (1 : 500) overnight at 4°C, washed 3 times by usage of TBST, and fostered by means of suiting horseradish peroxidase-labeled secondary antibodies (1: 1000) for 30 min at indoor temperature in a shaker. The bands were emerged by enhanced chemiluminescence (ECL). The relevant content of the target protein bands was profiled as the specific value of the grayscale value of the target protein bands to that of the GAPDH protein bands by semiquantitative analysis using the Image J software.

SPSS 23.0 statistical software was utilized for data analysis, and all data were subjected to a normal distribution and chi-square test. *X* ± *s* were utilized to express data obeying a Gaussian distribution. Independent samples *t*-test and one-way ANOVA was directed at comparison between two groups and multiple groups, respectively. Nonnormal distributions were denoted as median and extreme values, and the rank-sum test was accessed to compare differences disobedient the Gaussian distribution. *p* < 0.05 was expected statistically significant.

## 3. Results

### 3.1. Ang II Facilitates Podocyte Apoptosis

**Figure 1:**
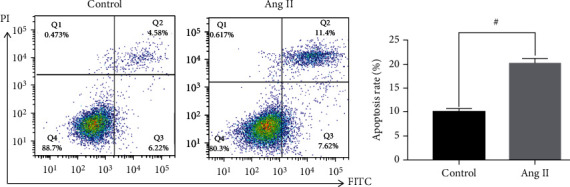
Ang II promotes podocyte apoptosis (*x* ± *s*, *n* = 3).

Podocytes were coped with 10-6 mol/L Ang II go by 48 h, and flow cytometry (FCM) was utilized to detect apoptosis. As illustrated in Figure 1, apoptosis of podocytes was significantly attracted by Ang II by comparison of controls group (^#^*p* < 0.05).

### 3.2. Effect of Ang II Intervention on the Expression Level of Cyr61 and TXNIP in Podocytes

**Figure 2:**
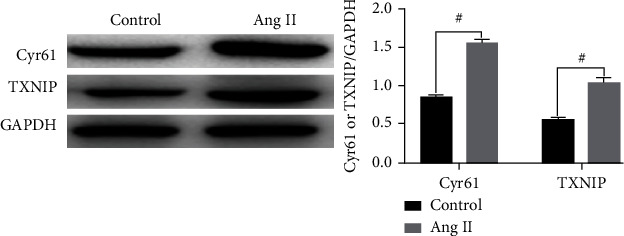
Ang II promotes the expression of Cyr61 and TXNIP (*x* ± *s*, *n* = 3).

The expression levels of Cyr61 and TXNIP were perceived through Western blot 48 h after intervention with 10-6 mol/L Ang II in podocytes. As depicted in Figure 2, the Cyr61 and TXNIP were expressively highly expressed following Ang II intervention be confronted with the matched group (^#^*p* < 0.05).

### 3.3. Affection of Ang II Intervention on the mRNA Expression Profile in Podocytes

**Figure 3:**
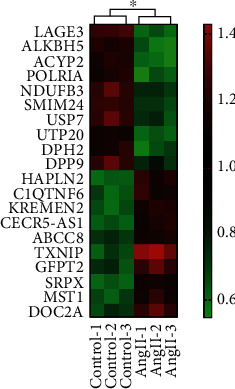
Heatmap displaying the top 10 most up- and downregulated mRNAs (*x* ± *s*, *n* = 3).

To inquire into the potential mechanism underlying the podocyte apoptosis induced by Ang II, alterations of mRNA expression profile after Ang II intervention in podocytes were further assessed using gene microarray technology. Using a 1.5-fold change as the cut-off value, we found that the expression of 588 mRNAs was altered, of which 248 were upside and 340 were downside. And TXNIP was the most significantly upregulated gene (Figure 3), and the 10 top significant expression genes are shown in Table 1.

### 3.4. Transfection Efficiency

**Figure 4:**
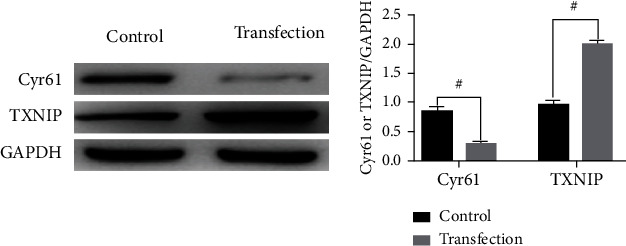
Expression level of Cyr61 and TXNIP proteins after transfection (*x* ± *s*, *n* = 3).

Cyr61 CRISPR/Cas9 KO plasmid and TXNIP activation plasmid were transfected according to the protocol of Cyr61 CRISPR/Cas9 KO plasmid and screened prior to cell collection and total cell protein extraction. Using Western blot detection level of the expression of Cyr61 and TXNIP in normal podocytes and podocytes with Cyr61 down and TXNIP overexpression, the outcomes of experiment revealed that Cyr61 were downregulated in transfection group significantly, and TXNIP were upregulated significantly in contrast with the comparison group (Figure 4) (^#^*p* < 0.05).

### 3.5. Cyr61 Knockdown Inhibits TXNIP Expression, While Overexpression of TXNIP Has No Significant Influence on the Expression of Cyr61

**Figure 5:**
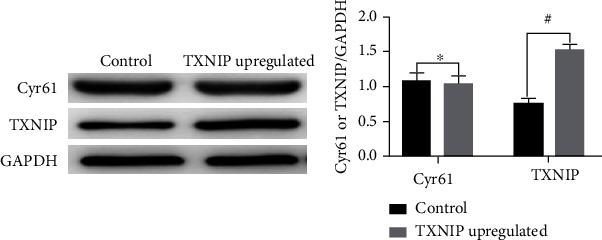
The effect of upregulating TXNIP on the expression of Cyr61 (*x* ± *s*, *n* = 3).

To verify the relationship between Cyr61 and TXNIP, changes in the expression levels of both proteins were explored by Western blot after knocking down Cyr61 and overexpressing TXNIP, respectively. The results demonstrated that knockdown of Cyr61 inhibited the expression of TXNIP (*p* < 0.05) compared with the control group (Figure 5), while overexpression of TXNIP had no available influence on the expression of Cyr61.

### 3.6. Effect of Cyr61 Knockdown on Ang II-Mediated Podocyte Apoptosis

**Figure 6:**
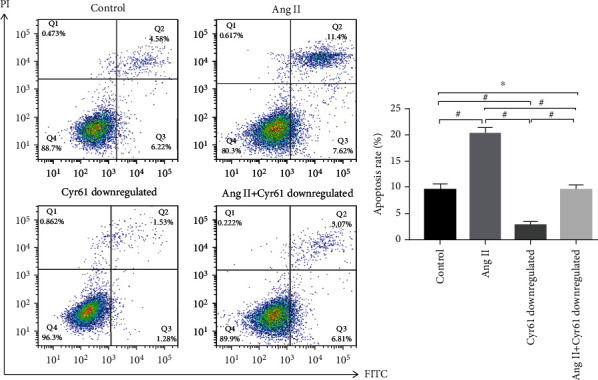
Effect of Cyr61 downregulation on Ang II-induced apoptosis of podocytes (*x* ± *s*, *n* = 3).

To further probe the character of Cyr61 in Ang II-induced podocyte apoptosis, we used CRISPR/Cas9 technology to knock down Cyr61 and observe the change of apoptosis rate of podocyte stimulated by Ang II. The results disclosed that, confronted with the comparison group, knockdown Cyr61 could reduce the apoptosis rate of podocyte (^#^*p* < 0.05). The rate of podocyte apoptosis with Ang II knockdown Cyr61 was expressively below the Ang II group (^#^*p* < 0.05), but had no virtual difference contradistinguished with the matched group (Figure 6) (^∗^*p* > 0.05).

### 3.7. Impression of Cyr61 Knockdown on Ang II-Induced Expression of TXNIP, Cyt-c, Bax, and Bcl-2 in Podocytes

**Figure 7:**
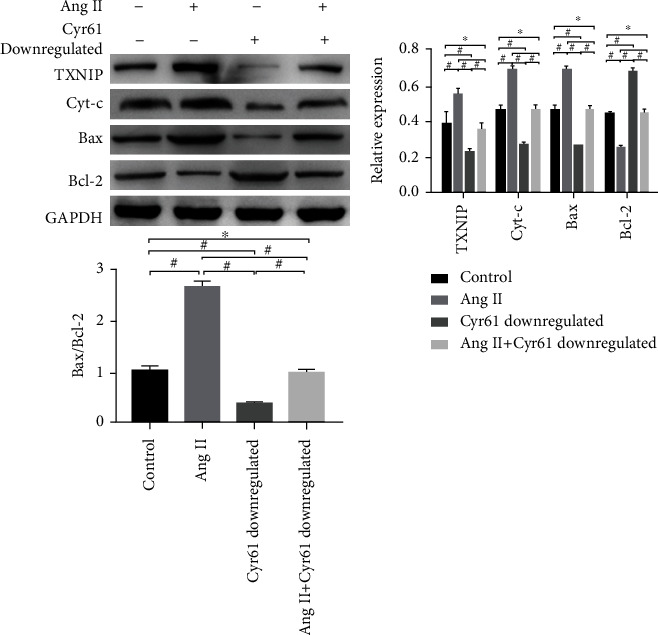
Effect of Cyr61 downregulation on the expression of level TXNIP, Cyt-c, Bax, and Bcl-2 induced by Ang II (*x* ± *s*, *n* = 3).

To further inquire into the role of Cyr61 in Ang II-induced podocyte apoptosis, the Western blotting were utilized to examine the alterations in TXNIP, Cyt-c, Bax, and Bcl-2 expression. Western blotting accounts determined that, contrasted with the comparison group, stimulation of podocytes using Ang II facilitated the expression of TXNIP, Cyt-c, and Bax (^#^*p* < 0.05), restrained the expression of Bcl-2 (^#^*p* < 0.05), and increased the Bax/Bcl-2 scale (^#^*p* < 0.05); knockdown of Cyr61 inhibited the expression of TXNIP, Cyt-c, and Bax (^#^*p* < 0.05), promoted Bcl-2 expression (^#^*p* < 0.05), and decreased the Bax/Bcl-2 ratio (^#^*p* < 0.05); there were no significant differences in TXNIP, CyT-C, Bax, Bcl-2, and Bax/Bcl-2 in Cyr61 podocytes stimulated by Ang II confronted with the comparison group (^∗^*p* > 0.05). The expression of TXNIP, CyT-C, and Bax was reductive (^#^*p* < 0.05), the expression of Bcl-2 was incremental (^#^*p* < 0.05), and the Bax/Bcl-2 was lessened (^#^*p* < 0.05) after Ang II stimulation knocked down Cyr61 podocytes (Figure 7).

### 3.8. Effect of TXNIP Overexpression on Ang II-Induced Podocyte Apoptosis

**Figure 8:**
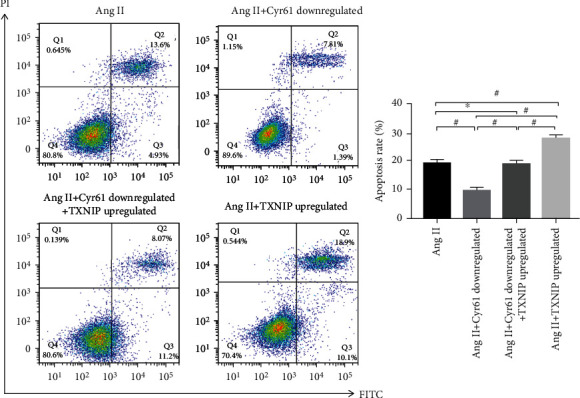
Effect of TXNIP upregulation on Ang II-induced apoptosis of podocytes (*x* ± *s*, *n* = 3).

The antecedent findings exposed that TXNIP expression was expressively elevated in answer to Ang II stimulation, and knockdown of Cyr61 inhibited TXNIP expression, implying that Cyr61 and TXNIP may be participated in Ang II-induced podocyte apoptosis. To further verify the character of Cyr61 and TXNIP in the podocyte apoptosis induced by Ang II, we overexpressed TXNIP using CRISPR/Cas9 technology and detected alterations in the apoptotic rate using flowmetry. Under Ang II stimulation, apoptosis was raising in the TXNIP overexpression group contradistinguished to the Ang II stimulation-only group (^#^*p* < 0.05), while there was no remarkable difference among the Cyr61 knockdown and TXNIP overexpression groups (^∗^*p* > 0.05). Besides, the apoptotic rate was enhanced in the TXNIP overexpression group confronted to the Cyr61 knockdown group (^#^*p* < 0.05), as well as in the Cyr61 knockdown + TXNIP overexpression group (^#^*p* < 0.05).  Figure 8 depicts mounted apoptosis in the TXNIP overexpression group compared to the Cyr61 knockdown + TXNIP overexpression group (Figure 8).

### 3.9. Effect of TXNIP Overexpression on Ang II-Induced Cyt-c, Bax, and Bcl-2 Expression Levels in Podocytes

**Figure 9:**
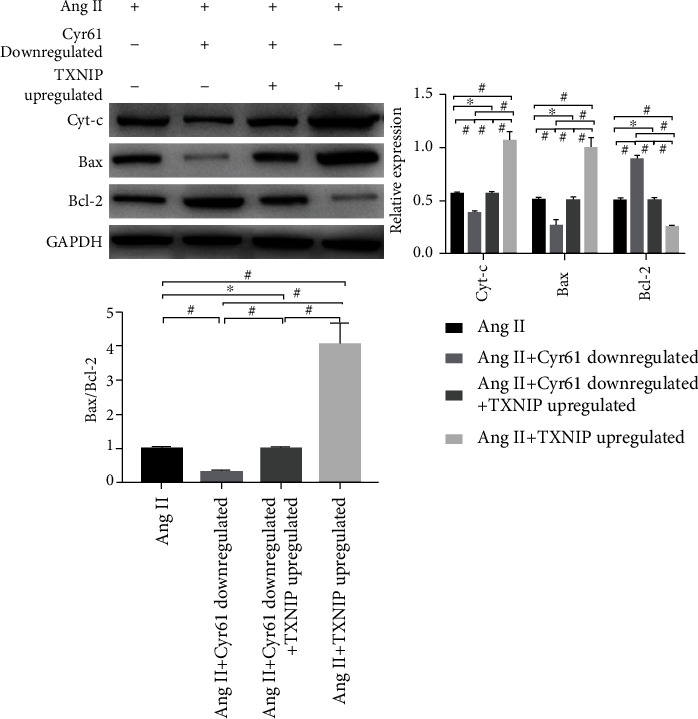
Effect of TXNIP upregulated on the expression of Cyt-c, Bax, and Bcl-2 following Ang II stimulation (*x* ± *s*, *n* = 3).

To investigate the identity of TXNIP in podocyte apoptosis induced by Ang I, changes in the expression levels of Cyt-c, Bax, and Bcl-2 were examined by way of Western blot. The outcomes demonstrated that under Ang II stimulation, the TXNIP overexpression group displayed increased expression of Cyt-c and Bax (^#^*p* < 0.05), shortened of Bcl-2 (^#^*p* < 0.05), and elevated Bax/Bcl-2 ratio (^#^*p* < 0.05) contrasted with the group activated with Ang II simply, whereas after simultaneous knockdown of Cyr61 and overexpression of TXNIP, there were no significant differences in Cyt-c, Bax, and Bcl-2 expression levels, as well as the Bax/Bcl-2 ratio (^∗^*p* > 0.05). Moreover, confronted with the group knockdown Cyr61, the group overexpressed TXNIP showed increased expression of Cyt-c and Bax (^#^*p* < 0.05) and lessened expression of Bcl-2 (^#^*p* < 0.05) and elevated Bax/Bcl-2 ratio (^#^*p* < 0.05).  Figure 9 delineates the affection of TXNIP upregulation on the expression levels of Cyt-c, Bax, and Bcl-2.

## 4. Discussion

Herein, we initially analyzed the apoptotic rate and alterations in Cyr61 and TXNIP expression levels in response to Ang II treatment of podocytes and detected changes in the RNA expression profile using gene microarray technology. Furthermore, CRISPR/Cas9 mechanics was used to knock down Cyr61 and overexpress the TXNIP gene in podocytes coped with Ang II. Flow cytometry used on detect apoptosis, while Western blot was employed to identify changes in Cyr61, TXNIP, Cyt-c, Bax, and Bcl-2 expression levels so as to probe into the underlying mechanism and role of Cyr61 and TXNIP in apoptosis in podocytes induced through Ang II.

Podocytes, highly differentiated epithelial cells of the visceral glomerular layer, are the organizers and managers of glomerular structures [15, 16]. They play an important role in glomerular filtration (GF), maintenance of glomerular basement membrane (GBM), glomerular capillary formation, and maintenance of glomerular capillary integrity, signaling, and other multiple counterfactuals [15]. Podocytes, together with glomerular capillary endothelial cells and glomerular basement membrane, constitute the glomerular filtration barrier. The cleavage septum formed between the peduncle of the podocyte is the final barrier of the glomerular filtration membrane, and its primary function is to form a protein-selective filtration barrier. The cleavage septum comprises several proteins, including nephrin, podocin, and transient receptor potential cation channel protein 6 (TRPC6). Disruption of these cleavage septum-associated proteins is implicated in the fusion and demise of podocytes and has also been revealed to be a critical link in the advance of proteinuric kidney disease [17, 18]. Podocyte damage from various causes eventually culminates in the replacement of podocytes by cicatricial tissue and extracellular matrix and results in the development of glomerulosclerosis and ultimately mediates the development of several chronic kidney diseases (CKD), including focal stage glomerulosclerosis, microscopic lesion nephropathy, membranous nephropathy, diabetic nephropathy, and lupus nephritis [16]. An in-depth study of the mechanism of podocyte injury may yield a new theoretical basis and avenue for the prevention and treatment of many glomerular diseases.

Ang II is one of the key RAS products and is known to play a crucial assignment in the administration of cardiovascular, renal inflammation, fibrosis, blood pressure, and renal hemodynamics [16–18]. Ang II serves distinct roles in different cells; for example, in vascular smooth muscle cells, it can promote their proliferation and lead to atherosclerosis [19]. Conversely, Ang II can operate apoptosis in podocytes, cardiomyocytes, and umbilical vein endothelial cells [20–22]. It is evident that the mechanism of action of Ang II is intricate, and an in-depth study of its mechanism of action is crucial to the prevention, diagnosis, and treatment of Ang II-related diseases.

The cysteinyl-rich protein 61 (Cyr61) remains with the secreted protein family of CCNs. It is a constituent of the extracellular matrix synthesized and excreted by endothelial cells, as well as a component of the cell membrane [19]. Earlier studies have established that it participates in various biological processes by regulating different signaling pathways. Furthermore, Cyr61 is essential during embryonic cardiovascular development, while in adulthood, it is closely related to inflammation, wound healing, injury repair, and related pathologies such as fibrosis and cancer [20]. The relevance of Cyr61 in different diseases has been extensively studied in recent years. Fan et al. [21] noted significantly higher levels of Cyr61 in the peripheral blood and lung tissue of patients with SLE-associated pulmonary hypertension compared to patients with nonpulmonary hypertensive SLE and healthy individuals. Li et al. [22] observed that Cyr61 was elevated in the very early stages of the disease by studying a rat matrix of renal ischemia-reperfusion injury. Shimura et al. [23] also concluded that urinary Cyr61 may be a noninvasive diagnostic criterion for colon cancer. Besides, Cyr61 is also closely associated with heart failure [24], coronary artery disease [25], and myocardial injury [26]. These findings infer that Cyr61 is jointed with the development of multiple diseases and may be a feasible target for the diagnosis and treatment of clinically relevant diseases.

Rodrigues-Díez et al. [27] found that the expression of Cyr61 was significantly upregulated and took part in the management of vascular smooth muscle cell proliferation after intervention with Ang II in rat vascular smooth muscle cells, suggesting that Cyr61 may be a downstream signaling molecule of Ang II. Our study revealed that the expression of Cyr61 was significantly elevated under the effect of Ang II; therefore, we postulated that Cyr61 might also participate in Ang II-induced podocyte injury. To verify this hypothesis, we administered Ang II to interfere with histocytes cultured in vitro, examined the altered RNA expression profile using gene microarray technology, and observed the effect of knocking down Cyr61 on Ang II-induced podocyte apoptosis. The results displayed that the expression of 588 mRNAs was altered after 48 h of Ang II action on the podocytes, of which 248 were upregulated and 340 were downregulated. As predicted, TXNIP was the most significantly upregulated gene. Meanwhile, Cyr61 protein expression was also increased, and the apoptotic rate of podocytes was significantly increased, while that of podocytes decreased after knocking down Cyr61 and overexpressing TXNIP. The above experimental results indicate that Cyr61 and TXNIP may be contained in Ang II-induced apoptosis of podocytes. By interfering with the expression of Cyr61, the occurrence of podocyte apoptosis could be effectively prevented.

Overexpression of TXNIP induces cell cycle arrest at G0/G1. There is an important role for TXNIP in apoptosis. Sun et al. [28] found that TXNIP expression was significantly reduced in hepatocellular carcinoma tissues. Additionally, through mitochondria-mediated ROS production and activation of MAPK pathways, TXNIP overexpression inhibited hepatocellular carcinoma cell proliferation. Yao et al. [29] reported that TXNIP was an important link in enterovirus 71-mediated apoptosis. Hou et al. [30] found that suppressing TXNIP effectively lowered lipopolysaccharide-induced apoptosis in umbilical vein endothelial cells. More importantly, Wang et al. [31] described that Ang II could mediate islet *β*-cell apoptosis by inducing TXNIP overexpression. Our study employed gene microarray analysis and Western blot to validate that intervention of podocytes with Ang II significantly promoted TXNIP expression and was closely associated with podocyte apoptosis. Consequently, knockdown of TXNIP significantly reversed Ang II-induced podocyte apoptosis.

Our further study revealed that knockdown of Cyr61 suppressed TXNIP expression, while overexpression of TXNIP had no valid impression on the expression of Cyr61. This finding signals that Cyr61 may be referred to Ang II-induced apoptosis in podocytes through the modulation of TXNIP. To test this conjecture, we knocked down both Cyr61 and overexpressed TXNIP in podocytes. Under Ang II stimulation, apoptosis was improved in the TXNIP overexpression group than the group stimulated with Ang II only in comparison. At the same time, there was no virtual difference in the apoptotic rate in the Cyr61 knockdown + TXNIP overexpression group. Apoptosis was elevated in the TXNIP overexpression group comparison with the Cyr61 knockdown group, although the apoptotic rate was increased in both the Cyr61 knockdown and TXNIP overexpression groups. These results provide robust evidence that Cyr61 plays a pivotal part in the podocyte apoptosis induced by Ang II and that Cyr61 can mediate the podocyte apoptosis induced by Ang II by promoting the expression of TNXIP.

The process of apoptosis, known as programmed cell death as well, is a complex process regulated by multiple pathways, among which the mitochondrial pathway is one of the key pathways regulating apoptosis. Cytochrome C (Cyt-c) is one of the basic components of the oxidative respiratory chain and plays a crucial part in the mitochondrial apoptotic pathway [32]. When cells are subjected to external stimuli, Cyt-c released from mitochondria into the cytoplasm can induce the activation of caspases, which in turn undergoes a cascade reaction to participate in apoptosis [33]. It has been shown that an increase in the Bax/Bcl-2 ratio can lead to the release of Cyt-c from mitochondria into the cell matrix [34] and that the dynamic balance of Bcl-2 family proteins, comprising the proapoptotic protein Bax and the antiapoptotic protein Bcl-2, located in the outer mitochondrial membrane [35], determines cell survival [36]. To investigate whether changes in Cyt-c and the Bax/Bcl-2 ratio are comprised in Ang II-mediated apoptosis in podocytes, the expression level of Cyt-c, Bax, and Bcl-2 was detected by Western blot after intervention with Ang II in podocytes herein. The outcomes showed that Cyt-c and Bax had a significantly raising expression, whereas that of Bcl-2 had a significantly dropped off, and the scale of Bax/Bcl-2 was boosted. The aforementioned results imply that Ang II-mediated apoptosis of podocytes may be associated with changes in Cyt-c and Bax/Bcl-2 ratio. Given the association between Ang II, Cyr61, and TXNIP in podocyte apoptosis, we further verified the relationship between Cyr61 and TXNIP with Cyt-c, Bax, and Bcl-2, and the results showed that knockdown of Cyr61 suppressed the expression of Cyt-c and Bax, promoted the expression of Bcl-2, and increased the Bax/Bcl-2 ratio; there was no statistical significance in the expression of Cyt-C, Bax, and Bcl-2 in podocytes with Cyr61 knockdown stimulated by Ang II (compared coped with Ang II only), the expression of Cyt-C and Bax was cut down, and the expression of Bcl-2 and the ratio of Bax/Bcl-2 was increased after treating podocytes that Cyr61 downregulated with Ang II. Under Ang II stimulation, the expression level of Cyt-c and Bax was incremental, that of Bcl-2 descending, and the ratio of Bax/Bcl-2 was declining in the TXNIP overexpression group in comparison with the Ang II stimulation only group, while there was no statistical significance in the expression of Cyt-c, Bax, and Bcl-2 in the Cyr61 knockdown + TXNIP overexpression group. Compared with the Cyr61 knockdown group, the Cyt-c and Bax expressed enhancive, that of Bcl-2 was decreased, and the Bax/Bcl-2 ratio was increased in the TXNIP overexpression group; similarly, Cyt-c and Bax had an ascending expression, that of Bcl-2 was degressive, and the ratio of Bax/Bcl-2 was expressed climbing in the Cyr61 knockdown + TXNIP overexpression group. Taken together, these results lead to the conclusion that Cyr61 may be comprised in podocyte apoptosis administration through the regulation of Cyt-c expression and Bax/Bcl-2 balance by Ang II through TXNIP.

Although our study initially confirmed the relationship and role of Cyr61 and TXNIP in Ang II-induced podocyte apoptosis, there were many shortcomings. Firstly, there was no negative control group during transfection, and we could not rule out the possibility that transfection reagents affected podocytes. Secondly, we simultaneously transfected two different genes in the podocytes, and it was not possible to effectively determine whether there was an interaction between the two transfection reagents. In addition, our study was limited to the influence of Ang II on podocyte apoptosis, while other cellular activities such as autophagy, cycle, and proliferation were not evaluated, nor were the molecular signaling mechanisms involved in apoptosis regulation, which may be the subject of our future study.

## 5. Conclusion

In short, TXNIP may be comprised in the podocyte's apoptosis induced by Ang II as a downstream signaling molecule of Cyr61, and blocking Cyr61 or TXNIP can significantly reduce the degree of Ang II-induced apoptosis in podocytes. Moreover, Cyr61 may mediate podocyte apoptosis through the involvement of TXNIP in the regulation of Cyt-c expression and Bax/Bcl-2 balance by Ang II. Therefore, inhibiting Cyr61 and its downstream-related signaling molecules in pathological conditions may be a patent strategy for the treatment of Ang II-related diseases.

## Figures and Tables

**Table 1:** The top 10 most significant up- and downregulated mRNAs.

*p* value	Gene symbol	Fold change (log FC)		Gene symbol	*p* value
		Upregulation	Downregulation		
5.08*E*-07	TXNIP	2.1059	-2.0432	LAGE3	0.0092
4.22*E*-07	HAPLN2	1.9081	-2.0415	ALKBH5	0.0028
3.47*E*-06	KREMEN2	1.8408	-2.0328	ACYP2	0.0001
1.05*E*-06	CECR5-AS1	1.8392	-1.9434	POLR1A	2.57*E*-05
5.45*E*-07	SRPX	1.8218	-1.8228	DPP9	0.0009
4.06*E*-06	C1QTNF6	1.8037	-1.8218	DPH2	6.25*E*-06
1.94*E*-06	DOC2A	1.7780	-1.7704	UTP20	5.15*E*-06
6.71*E*-06	GFPT2	1.7704	-1.7666	USP7	1.93*E*-05
0.0001	MST1	1.7560	-1.7127	SMIM24	3.14*E*-06
1.27*E*-04	ABCC8	1.5844	-1.6233	NDUFB3	0.0038

## Data Availability

The data used to support the findings of this study are included within the article.

## References

[1] Cheng H., Harris R. C. (2010). The glomerulus--a view from the outside--the podocyte. *The International Journal of Biochemistry & Cell Biology*.

[2] Gong X., Wu S., Chen M. (2017). Advances in the study of podocytosis. *Hunan Journal of Traditional Chinese Medicine*.

[3] Liu L. I. L., Mao Y. F. (2018). Research progress of targeted intervention therapy for podocytosis. *Chinese Journal of Clinical Physicians*.

[4] Lal M. A., Patrakka J. (2018). Understanding podocyte biology to develop novel kidney therapeutics. *Frontiers in Endocrinology*.

[5] Kumar R., Thomas C. M., Yong Q. C., Chen W., Baker K. M. (2012). The intracrine renin-angiotensin system. *Clinical Science*.

[6] Sobczuk P., Szczylik C., Porta C., Czarnecka A. (2017). Renin angiotensin system deregulation as renal cancer risk factor (review). *Oncology Letters*.

[7] Sparks M. A., Crowley S. D., Gurley S. B., Mirotsou M., Coffman T. M. (2014). Classical renin-angiotensin system in kidney physiology. *Comprehensive Physiology*.

[8] Zhang L., Ren Z., Yang Q., Ding G. (2016). Csk regulates angiotensin II-induced podocyte apoptosis. *Apoptosis*.

[9] Cardoso V. G., Gonçalves G. L., Costa-Pessoa J. M. (2018). Angiotensin II-induced podocyte apoptosis is mediated by endoplasmic reticulum stress/PKC-*δ*/p38 MAPK pathway activation and trough increased Na+/H+ exchanger isoform 1 activity. *BMC Nephrology*.

[10] Zhao M., Bai M., Ding G. (2018). Angiotensin II stimulates the NLRP3 inflammasome to induce podocyte injury and mitochondrial dysfunction. *Kidney Diseases*.

[11] Yang Y., Yang Q., Yang J., Ma Y., Ding G. (2017). Angiotensin II induces cholesterol accumulation and injury in podocytes. *Scientific Reports*.

[12] Wang J., Fu D., Senouthai S., You Y. (2019). Critical roles of PI3K/Akt/NF-*κ*B survival axis in angiotensin II-induced podocyte injury. *Molecular Medicine Reports*.

[13] Hartleben B., Gödel M., Meyer-Schwesinger C. (2010). Autophagy influences glomerular disease susceptibility and maintains podocyte homeostasis in aging mice. *The Journal of Clinical Investigation*.

[14] Mundel P., Reiser J., Borja A. Z. Mejía (1997). Rearrangements of the cytoskeleton and cell contacts induce process formation during differentiation of conditionally immortalized mouse podocyte cell lines. *Experimental Cell Research*.

[15] Endlich N., Siegerist F., Endlich K. (2017). Are podocytes motile?. *Pflügers Archiv*.

[16] Leeuwis J. W., Nguyen T. Q., Dendooven A., Kok R. J., Goldschmeding R. (2010). Targeting podocyte-associated diseases. *Advanced Drug Delivery Reviews*.

[17] Chuang P. Y., He J. C. (2009). Signaling in regulation of podocyte phenotypes. *Nephron Physiology*.

[18] Kwoh C., Shannon M. B., Miner J. H., Shaw A. (2006). Pathogenesis of nonimmune glomerulopathies. *Annual Review of Pathology*.

[19] Emre Y., Imhof B. A. (2014). Matricellular protein CCN1/CYR61: a new player in inflammation and leukocyte trafficking. *Seminars in Immunopathology*.

[20] Kim K. H., Won J. H., Cheng N., Lau L. F. (2018). The matricellular protein CCN1 in tissue injury repair. *Journal of Cell Communication and Signaling*.

[21] Fan Y., Zhao J., Qian J. (2019). Cysteine-rich protein 61 as a novel biomarker in systemic lupus erythematosus-associated pulmonary arterial hypertension. *Clinical and Experimental Rheumatology*.

[22] Li C., Zhao L., Wang Y. (2019). Cysteine-rich protein 61, a specific ultra‐early biomarker in kidney ischemia/reperfusion injury. *Nephrology*.

[23] Shimura T., Iwasaki H., Kitagawa M. (2019). Urinary cysteine-rich protein 61 and trefoil factor 3 as diagnostic biomarkers for colorectal cancer. *Translational Oncology*.

[24] Zhao J., Zhang C., Liu J. (2018). Prognostic significance of serum cysteine-rich protein 61 in patients with acute heart failure. *Cellular Physiology and Biochemistry*.

[25] Deng J., Qian X., Li J., Li Y., Li Y., Luo Y. (2018). Evaluation of serum cysteine-rich protein 61 levels in patients with coronary artery disease. *Biomarkers in Medicine*.

[26] Klingenberg R., Aghlmandi S., Liebetrau C. (2017). Cysteine-rich angiogenic inducer 61 (Cyr61): a novel soluble biomarker of acute myocardial injury improves risk stratification after acute coronary syndromes. *European Heart Journal*.

[27] Rodrigues-Díez R., Tejera-Muñoz A., Esteban V. (2022). CCN2 (cellular communication network factor 2) deletion alters vascular integrity and function predisposing to aneurysm formation. *Hypertension*.

[28] Sun Q., Wang B., Wei W. (2022). ITCH facilitates proteasomal degradation of TXNIP in hypoxia- induced lung cancer cells. *Thoracic Cancer*.

[29] Yao C., Hu K., Xi C., Li N., Wei Y. (2019). Transcriptomic analysis of cells in response to EV71 infection and 2A(pro) as a trigger for apoptosis via TXNIP gene. *Genes Genomics*.

[30] Hou X., Yang S., Yin J. (2019). Blocking the REDD1/TXNIP axis ameliorates LPS-induced vascular endothelial cell injury through repressing oxidative stress and apoptosis. *American Journal of Physiology. Cell Physiology*.

[31] Wang J., Feng Y., Huo H. (2019). NLRP3 inflammasome mediates angiotensin II-induced islet *β* cell apoptosis. *Acta Biochimica et Biophysica Sinica*.

[32] Wu L. F., Yang A. J., Liu H., Wang Y. N. (2010). Advances in mitochondrial regulation of apoptosis. *China Agricultural Bulletin*.

[33] Wang Y. J., Deng W., Zhang P. F. (2012). Advances in the study of cytochrome C and apoptosis. *Advances in Animal Medicine*.

[34] Philchenkov A. (2004). Caspases: potential targets for regulating cell death. *Journal of Cellular and Molecular Medicine*.

[35] Krajewski S., Tanaka S., Takayama S., Schibler M. J., Fenton W., Reed J. C. (1993). Investigation of the subcellular distribution of the bcl-2 oncoprotein: residence in the nuclear envelope, endoplasmic reticulum, and outer mitochondrial membranes. *Cancer Research*.

[36] Ola M. S., Nawaz M., Ahsan H. (2011). Role of Bcl-2 family proteins and caspases in the regulation of apoptosis. *Molecular and Cellular Biochemistry*.

